# A Qualitative Study to Explore the Experiences of Older People Utilising Outpatient Healthcare Services from a Teaching Hospital in Ghana

**DOI:** 10.1155/2020/2593795

**Published:** 2020-10-03

**Authors:** Peter Adatara, Philemon Adoliwine Amooba

**Affiliations:** ^1^Department of Nursing, School of Nursing and Midwifery, University of Health and Allied Sciences, Volta Region, Ho, Ghana; ^2^Department of Nursing, College of Health Sciences, PMB, KNUST, Kumasi, Ghana

## Abstract

**Background:**

Empirical evidence suggests that when older people are provided with quality and affordable healthcare services, their health status can be improved. However, in low- and middle-income countries, healthcare services may not be fully resourced, leading to difficulties for older people utilising those services. There is a paucity of research studies regarding the experiences of older persons accessing healthcare services in sub-Saharan Africa including Ghana.

**Aim:**

The purpose of this study was to explore and describe the experiences of older people utilising outpatient healthcare services from a Teaching hospital in Ghana.

**Methods:**

This study adopted a descriptive qualitative approach to describe the experiences of older persons utilising outpatient healthcare services. Participants included a total of twelve older people between the ages of 60 and 80 years who utilised outpatient healthcare services at the hospital. A semistructured interview guide was used to conduct interviews with participants, and their views were analysed into descriptive themes.

**Results:**

The results in this study showed that the participants experienced the following in utilising outpatient healthcare services: long waiting hours in accessing outpatient healthcare, poor attitudes from health staff, lack of drugs from the healthcare facility, inadequate information from health staff in the healthcare facility, lack of specialist care at the healthcare facility, and high confidence and trust in the efficacy of the orthodox healthcare.

**Conclusion:**

The findings from this qualitative study demonstrated that the participants encountered some challenges in accessing outpatient healthcare services. There is the need for government and other stakeholders to address the challenges encountered by older people in accessing healthcare in order to facilitate the utilisation of healthcare among older persons for better health outcomes.

## 1. Background

The utilization of health care services by older people (people who are aged 60 years or over) plays an important role in the health and wellbeing of the aged in society. The development of every country largely depends on how well its population is taken care of by the healthcare system [1]. Research shows that the world population of people who are aged 60 years and above has increased over the years, and there is the need for healthcare providers to plan to provide healthcare services that meet the specific needs of the increasing number of older people [2].

In Ghana, evidence shows that the proportion of persons aged 60 years or older is on the rise. The Ghana Statistical Service report indicates that the population of people aged 60 years and above constituted 6.7% of Ghana's total population [3]. Evidence shows that Ghana has one of the highest proportions of older persons in sub-Saharan Africa [3]. Unfortunately, the increase in the number of older persons has not had a corresponding increase in social and health care support systems [2]. Due to the ageing population, there is a pressure on the Ghana government and all stakeholders to work hard to improve the health care systems to facilitate access and utilization of health care services by older people in order to promote, prevent, and treat chronic diseases such as diabetes, hypertension, dementia, and other diseases [1]. It has been reported by previous studies conducted in Ghana that there are increasing levels of occurrence of noncommunicable and other chronic diseases such as hypertension, diabetes mellitus, and cancers among older people [4]. Despite the increasing levels of occurrence of such diseases, previous research studies reported that there is poor utilisation of outpatient health services among older people in Ghana [4]. Also, recent reports from the Ghana Health Service indicate that older people in Ghana face increasing higher rates of mortality and morbidity due to lack of a well-developed healthcare system to deal with the special needs of older people [5].

In Ghana, several initiatives and policy interventions have been put in place to facilitate access and utilization of health care services by older persons [6]. For instance, in 2008, the government of Ghana introduced the National Health Insurance Scheme (NHIS) which exempted persons aged 70 years and above from paying for subscription [7]. Under the NHIS, the aged are entitled to access healthcare services from any health facility without paying any money.

In spite of the introduction of the NHIS and other social protection programme policies to facilitate access and utilization of healthcare services by older persons, previous research studies in Ghana show that older persons still do not patronize health care [2, 8–10]. The studies suggest that older persons mostly do access alternative medicine such as seeking care from traditional herbalists or prayer camps when they are sick [4, 11]. The underutilisation of orthodox health care services among older persons is a worrying issue for healthcare providers and other stakeholders that need attention. However, little is understood about the experiences of older people utilising outpatient healthcare services, and knowing more about this could shed light on the underutilisation rates. It is in the light of the above that this study seeks to explore and describe experiences of older people utilising outpatient healthcare services at a referral health facility in the Volta Region of Ghana where majority of the older people access healthcare.

## 2. Methods

### 2.1. Research Design

A descriptive qualitative approach was used to examine the experiences of older persons utilising outpatient healthcare services. A qualitative approach was chosen for this study due to the fact that it enables the researcher to collect rich and varied opinions from the participants in detail [12].

### 2.2. Research Setting

This study was carried out at a referral health facility in the Volta Region of Ghana where majority of the older people access healthcare. The health facility provides both outpatients and inpatient healthcare services. It provides outpatient healthcare services such as diagnosis and treatments for patients who do not need admission into a hospital ward. The hospital is a 306-bed capacity health facility that also provides inpatient health services in six main clinical departments including internal medicine, surgical, obstetrics and gynaecology, child health, and public health. Other services that are provided in the health facility include intensive care units, neurological, dialysis, and cardiovascular units. The hospital, however, does not have a geriatric unit separately for older people. Older persons with geriatric conditions would have to access healthcare with other age groups in the health facility.

The health facility has a total of twenty medical doctors comprising of consultants, senior specialists and specialists, residents, and medical officers providing healthcare to over 100 patients and clients on monthly basis. The hospital also has fifty-five nurses of various categories providing nursing care to patients. Apart from doctors and nurses, there are other paramedical staffs such as pharmacists, medical laboratory scientists, radiographers, and support staff providing care. The hospital does not have geriatric specialist doctors or nurses; it is the general medical practitioners who attend to all outpatients including the older persons.

### 2.3. Study Participants

The participants for this study were a total of twelve older persons aged between 60 and 80 years who were conveniently selected for semistructured interviews. A convenient purposive sampling strategy was used to select participants who were available and willing to participate in the study. Both men and women were included in the study. Interviews with participants were conducted at their homes rather than at the hospital in order not to cause any inconvenience.

### 2.4. Recruitment of Participants

The researchers visited the outpatient department of the hospital to recruit participants for this study. The potential participants were identified by the OPD nurses who had been informed by the researcher regarding the aim of this study. The purpose of the study was explained to the participants by the nurses working at the health facility. Information sheets which explained the purpose and rationale for the study were given to study participants who could read and understand, and verbal explanation was also given to participants who could not read to enable them decide freely whether or not they will be willing to participate in the research study. The researchers told participants that participation was voluntary, and they have the right to change their mind and withdraw at any time within a two-week period without giving a reason and without their participant rights being affected. After learning the purpose of the study, those who agreed to participate in the study were asked to choose the date, venue, and time appropriate for them for the interview. This was done to avoid inconveniencing the participant. The researchers shared with the agreed participants their telephone numbers addresses to follow up for the interviews. Participants were told they would be audio taped but their anonymity and confidentiality will be assured.

### 2.5. Data Collection

In this study, the researcher employed semistructured interview schedules, field notes, and self-reflective journals to collect relevant and rich data for the study. A one-on-one interview was conducted with each of the participants, which allowed the researchers and participants the opportunity to ask follow-up questions to clarify doubts [13]. The interview guide was pretested with two participants to identify ambiguous questions. Interviews were conducted in the English, Ewe, and Twi languages, depending on the preferences of each participant. There was no need for a translator since the researchers could speak any of the three languages. Each interview lasted between 45 and 60 minutes. All interviews were digitally recorded, following participants' consent.

### 2.6. Data Analysis

The researchers adopted a descriptive thematic analytical approach. The analysis of the data followed the guide proposed by [14]. The first step was reading the transcripts to become familiar with the data. The transcripts were read many times while taking down notes. The next step was that the data were coded using the NVivo version 12 software and initial codes were generated from reading the transcripts. The coding was done descriptively, by attending to the research questions of this study. After generating many codes, the researchers searched for themes. Codes of similar concept were put together into themes. Six themes emerged from the analysis.

### 2.7. Ethical Considerations

The approval for this study was granted by the University of Southampton's Ethics Research and Governance Organisation (ERGO). Also, institutional approval from the Teaching Hospital in which participants were recruited was sought before the start of data collection. The participants were given the chance to decide voluntarily whether to participate in the study or refuse, without risking any penalty or prejudicial service or treatment [15]. Participants' information leaflets, which contain all the information regarding the study before the participants gave consent, were explained for all the participants. Participants were told that measures would be put in place to ensure that their identity and all information concerning them are protected. Participants were given the assurance that they could withdraw from the study anytime, and it will not be used against them when they are seeking healthcare or any related health issue. After explaining the purpose of the study, participants were given informed consent forms which were duly filled and signed by them. The semistructured interviews were conducted using their identity numbers instead of their names to ensure the confidentiality of participants. Participants were identified with codes to ensure anonymity.

### 2.8. Trustworthiness

In this study, rigour was ensured by detailed documentation of all antecedent stages and processes to data collection, a detailed account of participant selection and approach, a clear transcription and coding approach using Braun and Clarke guidelines which include reading the transcripts several times to become familiar with data, generating initial codes from the data, searching for themes, reviewing themes identified, defining themes, and writing up themes [14]. A vivid and consistent presentation of the results was also provided in the analysis of data in this study. Similarly, thick description of the research setting as well as the context was also done to facilitate logic of process, audit trail/dependability audit [16]. Moreover, strategies that ensured reliability of the study also include dense descriptions of the research approaches and methods as well as the findings, code, and recode process of data analysis. Also, the interview guide was piloted on a selected group of people with similar characteristics to allow adjustment of the questions. The researchers had prolonged familiarisation with older persons to ensure in-depth knowledge of the themes that came out from the interview and data analysis. Data transcriptions and coding were done by two researchers with rich experience in qualitative studies to ensure that the right challenges and expectations were reported. The researchers went back to older persons to find out if themes formulated represented their opinions of which all of them confirmed that themes truly reflected their views presented during the interviews.

## 3. Results

Participants were between 60 and 80 years. Four of the participants were farmers, two were artisans, and two were traders, whiles three were receiving a pension. For confidentiality and anonymity, identification numbers such as P1-P11 were used where “P” denotes participant.

### 3.1. Themes That Emerged from the Analysis

The older people expressed various experiences and opinions regarding their use of outpatient healthcare services at a referral health facility in the Volta Region. The following are the experiences of older persons utilising outpatient healthcare services at a referral health facility in the Volta Region:

- Long waiting hours to access outpatient healthcare
- Poor attitudes from health staff
- Lack of drugs from the healthcare facility
- Inadequate information from health staff in the healthcare facility
- Lack of specialist care at the healthcare facility
- High confidence and trust in the efficacy of the orthodox healthcare

### 3.2. Theme 1: Long Waiting Hours to Access Healthcare

The majority of participants expressed their experiences and frustrations of long waiting hours to access healthcare in healthcare facilities as a result of joining long queues for treatment at the outpatient departments in healthcare facilities.

“I am always joining long queues with the young and energetic people. There are few nurses attending to both the elderly and the young ones. They keep us in a long queue; keep us waiting before later seeing the doctor”. [P2].

*“I was disappointed when I went to the hospital one Sunday when I had asthmatic attack. I got to the hospital around 9: 00* AM *and in spite of the critical nature of my condition, I had to wait for an hour to be attended to because the nurses were only two on duty and they were all busy working on some patients”. (P11).*

Participants indicated that because of the long queues at public healthcare facilities, the majority of the participants in the study preferred to use the private facilities to public healthcare facilities.

“If I had having enough money, I would have gone to the private health facility and not here because at the private health facility as an old person like me they will attend me quickly and I will go back home early. There are no long queues there”.

The long waiting hours in queues have resulted in some older persons seeking to treat themselves with over the counter medications instead of reporting to the hospitals for treatment. The participants felt because they are old and very weak, they should have been the first people to be attended to before the younger people who are still strong.

“It is not easy when you think of going to the hospital. All that comes to mind is the long queues that will be waiting for you when you get there. Sometimes I just go to the drug store or the local chemist shop to get something for myself instead. But I think it is not the best”. [P 8].

Some of the participants indicated that they usually try other sources of treatment in the house without going to the hospital because of the frustrations they experience in hospitals. However, they visit hospitals when there are complications in their conditions:

“As for me, I only came here when my blood pressure was very high and I collapsed in the house. I was only buying herbs from the herbal shops at my place to take until my condition became bad”. [P 3].

Participants reported that long hours of waiting to access healthcare at the Outpatient Department usually affect only older people who do not know any worker in hospital. But, those who have relatives or friends who are working as nurses are usually assisted to access care very early.

“As for patients who have relatives or friends who are nurses they go through the back door to get the same service that those of us in the queue are also seeking just because their relations are staff members of the hospital”. (P5).

Although sometimes the above incidence does happen in public healthcare facilities, it is often done in secret, because it is unethical and it is against health facility rules and any health personnel that is caught in that act is punished.

### 3.3. Theme 2: Poor Attitudes of Staff and Poor Quality of Services at the Healthcare Facilities

Most of participants reported poor attitude of some nurses at the outpatient department in many healthcare facilities, especially in the public hospitals.

“I stopped going to the hospital because exactly a month ago when I visited there to seek treatment, the nurse shouted at me because I was talking to someone when she was making an announcement”. (P10).

Some of the participants reported that health personnel were overwhelmed by the number of patients accessing healthcare services during weekends and public holidays which increase the suffering of older persons.

“I think the nurses are always overwhelmed by the number of patients accessing healthcare services during weekends and public holidays. I realised that when I visited the hospital one Saturday, the nurse was the only one on duty and yet we the patients were many”. (P2)

The majority of participants reported that the healthcare services that were provided at private health facilities were much better than the services at the public health facilities.

“I think the private ones pay more attention, the reception they give you is better when you look at the attitudes of the nurses toward patients. That is why I do go there more regularly than the public ones like this hospital”. (P8).

### 3.4. Theme 3: Lack of Drugs from the Healthcare Facility

Some of the participants also reported lack of medicine and other consumables at the health facilities:

“After waiting for hours to see a doctor I was only given three sachets of paracetamol tablets, and later I was told to get haemoglobin drug in town because it was not available”. (P8).

“I must say that one of the problems we mostly encounter anytime we visit the health facility to access care is lack of essential drugs. Many at times, the drugs that the doctor prescribed for you, you will not get it in the hospital”. (P2).

Some of the participants attributed the lack of medicine and other consumables at the health facility to National Health Insurance. They indicated that because the National Health Insurance policy exempted older persons who are 70 years and above from paying subscription fee, healthcare providers sometimes refuse to supply all the drugs they need.

“I was not given most of the drugs that were prescribed for me when I visited the hospital. And I know it is because the National Health Insurance policy exempted older persons who are 70 years and above from paying subscription fee, they refused to give me all the drugs”. (P4).

Moreover, some of the participants reported that when they visited the hospital, they were only given drugs that are not expensive. But the drugs that were prescribed for them which were expensive were not supplied to them. One of them had this to say:

“I was only given drugs which were not expensive. But the expensive ones, I was asked to go and buy them from pharmacy shops in town”. (P8).

### 3.5. Theme 4: Inadequate Information from Health Staff

The majority of the older persons pointed out that they received inadequate information from nurses in the outpatient department and other departments in hospitals. Older persons were of the view that nurses were usually too busy to talk with them:

“Nurses did not explain issues into details when taking care of me. Most of the time they will come and say, Ma'am I want to do this and that for you. Maybe it is because they don't have time. Not much explanation is given on the needs and how important that task will affect my health. I really think it is the lack of time in the hospital and workload”. (P10).

Inadequate information made older persons wander within hospital environments in a bid to find various departments within the hospital for services. Older persons were of the view that they should not be made to wander like younger clients.

“Not enough information is given. Nurses will usually tell you to go and do this and that investigation but sometimes we don't even know where those places are. You have to roam around the whole place sometimes before finding places for these services”. (P11).

Some older persons thought there should be reception desks at outpatient department of hospitals similar to what can be found in many organizations in Ghana. They wished they could be given necessary information at these proposed receptions without having to ask for such information from nurses:

“The issue for me is the information in our hospitals. Some workers should be there to explain things to us to understand well. Information is necessary to know what to do in the hospital environment. It is not very nice to be sent around any how in the hospital when you don't even have adequate information”. (P2).

### 3.6. Theme 5: Lack Specialist Care at the Healthcare Facility

Another major challenge encountered by older persons as reported by participants was lack of specialists in geriatric care in the hospitals. This has often made older persons move from one health facility to another in search of specialist in geriatric care.

“We have being suffering in this country as old people. We don't have doctors and nurses who have been trained to take care of old people. So, as an old person when you are sick and go to hospital, the young doctors and nurses will treat us like other people. But we have special problems that need special attention”. (P. 11).

Participants were of the view that because they do not have geriatric specialists to handle their health condition, old persons with multiple diseases are moved from one unit to another in the hospital.

“Sometimes you go to the hospital to join a long queue and when it is your turn to see the doctor, the nurse will tell you to go another consulting room to see another doctor because this doctor does not attend to people with this condition”. (P2).

### 3.7. Theme 6: High Confidence and Trust in the Efficacy of the Orthodox Healthcare

The findings of this study demonstrate that although older persons had negative experiences such as long waiting hours to access healthcare in healthcare facilities, poor attitudes of staff and poor quality of services, and inadequate information from health staff in healthcare facilities, they had high confidence and trust in the efficacy of the orthodox drugs.

“*We have high confidence in orthodox drugs and treatment because they are better than herbal drugs. When we take the drugs from the hospitals we feel fine as compared to herbal preparations”. (P5).*

Participants indicated that given the opportunity, the majority of them would prefer to access health care from the orthodox healthcare facilities because of efficacy of the orthodox drugs.

“The orthodox drugs are good. If I have money I will never access healthcare services anywhere because the drugs work well for me”. (P7).

Participants indicated that although they high confidence and trust on the efficacy of the orthodox drugs and treatment because of financial and other reasons, they sometimes seek treatment from other healthcare providers.

“As for me, I have high confidence and trust on the efficacy of the orthodox drugs and treatment and always want to access healthcare from orthodox healthcare facilities rather than traditional and alternative medicines but because of financial and other challenges, I have to sometimes seek healthcare from other healthcare providers”. (P10).

## 4. Discussion

This study sought to explore the experiences of older persons utilising outpatient healthcare services at the Ho Teaching Hospital, Ghana. The results showed that older people experienced the following in accessing outpatient healthcare services: long waiting hours to access healthcare at the outpatient departments of healthcare facilities, poor attitudes of staff and poor quality of services at the healthcare facilities, inadequate information from health staff in healthcare facilities, lack of specialist care at the healthcare facilities, and high confidence in the efficacy of orthodox medicine to cure their illness. The findings of this study confirm the results of previous studies that were conducted in other low-income countries [4, 17]. For instance, it was reported in a study conducted in Nigeria on the experiences of the elderly in seeking healthcare services in a district hospital in Lagos that older persons experienced frustrations such as waiting for longer hours to access care, poor attitudes of nurses, and inadequate information from nurses on their disease conditions in accessing health care services [17]. Similarly, it was reported in Cape Town, South Africa, that participants experienced poor doctor-patient communication, poor nurse-patient communication, and long waiting hours to access healthcare services [18]. The findings are also consistent with research studies conducted in other regions of Ghana. For example, it was reported in a study conducted in the Asante Akyem North District that older people experience long waiting hours in health facilities to access healthcare services [8]. The findings highlighted above imply that older persons go through stressful situations in accessing outpatient health services in Ghana. What probably contributes to the long waiting hours by older persons in accessing healthcare services by older persons might be linked to the fact that there are no specific consulting room assigned to only older persons at the outpatient department. Older persons are expected to join queues with other age groups to access healthcare service. Although old people join queues to access healthcare in other public health facilities in Ghana, considering the fact that they are old and sometimes look very weak when accessing healthcare, it would be a good idea to separate them from other age groups. There is the need for healthcare providers to separate older persons from other age groups to reduce the waiting hours, since many of them come to a health facility with chronic noncommunicable diseases such as hypertension and diabetes making them very weak when they are to wait for long hours before accessing healthcare.

Additionally, the findings of this study showed that older people experienced lack of gerontology specialist care at the healthcare facilities. In Ghana, there are no gerontology specialist doctors and nurses caring for older persons. Biritwum et al. reported similar findings in their study that although Ghana is experiencing demographic transition with an increased in the older population, there is no preparation from the healthcare sector of Ghana to meet the needs of older persons [4]. They reported in their findings that there is no single trained geriatric doctor or nurse in Ghana to attend to healthcare needs of older persons, and this has often made older persons to move from one healthcare facility to another in search of healthcare services that meet their needs. Similarly, it was reported in a study in South Africa that doctors and nurses do not want to specialize in gerontology, because they felt there are no monetary rewards in the field of gerontology [18]. This is probably due to the fact that doctors and nurses do a part-time job to earn additional income, specialising in gerontology will not afford them the opportunity to do a part-time job because the private health facilities do not have gerontology departments. The lack of specialised care in gerontology in most developing countries have has often compelled older persons to resort to seeking healthcare from general medical and nursing practitioners who sometimes do not appreciate the multidisciplinary nature of gerontological care [19]. Moreover, the lack of specialist care in Ghana and other African countries demonstrates that developing countries need to train healthcare specialists in gerontology to take care of older persons.

The results of this study demonstrate that although older persons had negative experiences such as long waiting hours to access healthcare in healthcare facilities, poor attitudes of staff and poor quality of services, and inadequate information from health staff in healthcare facilities, the majority of the older persons still patronize orthodox healthcare and would only resort to seeking care from other traditional health practitioners if they cannot afford the services of orthodox healthcare. These clearly demonstrate that if healthcare managers implement aged-friendly healthcare delivery services that address specific needs of the older people in their facilities, the majority will access their care which will lead to better health outcomes for older people.

### 4.1. Study Limitations

This study was mainly carried out at Ho Teaching Hospital. Experiences of older persons utilising outpatient healthcare services from district hospital and primary healthcare facilities were not explored. Although exploring the experiences of older persons utilising outpatient healthcare services from district hospital and primary healthcare facilities might not have affected or changed the findings of this study, it would have given an understanding of the experiences of older people utilising outpatient health services from rural settings in Ghana.

The study was also limited to older people's experiences of utilising outpatient healthcare services, but older people who utilised inpatient healthcare services were not explored. Exploring the experiences of older persons' utilising inpatient healthcare services would have given a holistic view on older persons' experiences.

## 5. Conclusion

In conclusion, it came to light in this study that older persons in Ghana experience long waiting hours to access healthcare in healthcare facilities, poor attitudes of staff and poor quality of services at the healthcare facilities, inadequate information from health staff in healthcare facilities, and lack of specialist care at the healthcare facilities. There is the need for the government of Ghana and other stakeholders to address the above concerns of older persons to enable them to utilise healthcare services from orthodox health facilities for better health outcomes.

### 5.1. Recommendations

The following recommendations were made for policy, practice, and further studies.

### 5.2. Improve Healthcare Practice

The following recommendations are made to improve healthcare practice based on the results of this study:

- Healthcare providers should provide adequate and clear information to older persons regarding treatment information, diagnoses, and instruction of drug dosages
- Healthcare providers should educate their healthcare staff especially nurses who are frontline service workers and are mostly with patients and clients to respect older persons when they come to access healthcare services at their facility. Workshops should also be organized for practicing nurses and nursing leaders on the care of older people

### 5.3. Further Research

It is recommended that further research study be carried out to explore the experiences and challenges of older persons utilising health care services from district and primary health care facilities. A follow-up research study is needed to holistically explore the experiences and challenges of older persons in utilising inpatient healthcare services using a quantitative approach to cover a large number of people in order to generalise results.

## Data Availability

The transcripts from which this manuscript was developed are available on request from the corresponding author.
